# miDruglikeness: Subdivisional Drug-Likeness Prediction Models Using Active Ensemble Learning Strategies

**DOI:** 10.3390/biom13010029

**Published:** 2022-12-23

**Authors:** Chenjing Cai, Haoyu Lin, Hongyi Wang, Youjun Xu, Qi Ouyang, Luhua Lai, Jianfeng Pei

**Affiliations:** 1 Center for Quantitative Biology, Academy for Advanced Interdisciplinary Studies, Peking University, Beijing 100871, China; 2 BNLMS, State Key Laboratory for Structural Chemistry of Unstable & Stable Species, College of Chemistry and Molecular Engineering, Peking University, Beijing 100871, China; 3 Infinite Intelligence Pharma, Beijing 100083, China; 4 The State Key Laboratory for Artificial Microstructures and Mesoscopic Physics, School of Physics, Peking University, Beijing 100871, China; 5 Research Unit of Drug Design Method, Chinese Academy of Medical Sciences (2021RU014), Beijing 100871, China

**Keywords:** subdivisional drug-likeness prediction, active learning, ensemble learning, graph neural network

## Abstract

The drug development pipeline involves several stages including in vitro assays, in vivo assays, and clinical trials. For candidate selection, it is important to consider that a compound will successfully pass through these stages. Using graph neural networks, we developed three subdivisional models to individually predict the capacity of a compound to enter in vivo testing, clinical trials, and market approval stages. Furthermore, we proposed a strategy combing both active learning and ensemble learning to improve the quality of the models. The models achieved satisfactory performance in the internal test datasets and four self-collected external test datasets. We also employed the models as a general index to make an evaluation on a widely known benchmark dataset DEKOIS 2.0, and surprisingly found a powerful ability on virtual screening tasks. Our model system (termed as miDruglikeness) provides a comprehensive drug-likeness prediction tool for drug discovery and development.

## 1. Introduction

Computational drug-likeness prediction aims to assess the chance for a compound to become a marketed drug and is one of the essential metrics for screening drug candidates. Early drug-likeness predictions were made by extracting rules based on physicochemical properties. Lipinski et al. proposed ‘the Rule of 5’ (Ro5) to exclude compounds with poor absorption or permeation, which are unbeneficial for drug-likeness [1]. The Ro5 states that a compound is likely to have poor absorption or permeation when it has more than 5 hydrogen-bond donors, more than 10 hydrogen-bond acceptors, a molecular weight greater than 500 Da, and a calculated octanol–water partition coefficient greater than 5. Ghose et al. proposed an additional physicochemical property, molar refractivity, to complete the Ro5 [2]. Similarly, Veber et al. included the number of rotatable bonds and the polar surface area to account for molecular flexibility [3]. Although rule-based methods are convenient to implement, they are not applicable to all drug molecules, because they are based on simple criteria. According to a previous study, 16% of 771 oral drugs from the ChEMBL database of bioactive molecules with drug-like properties violate at least one component of the Ro5, and 6% violate more than two [4]. For a more quantitative estimation of drug-likeness, Bickerton et al. introduced the Quantitative Estimation of Drug-Likeness (QED) score [4] based on desirability functions fitted to the distributions of eight physicochemical properties, including properties used by Veber filters [3], the number of aromatic rings, and the number of structural alerts. QED not only outperforms the Ro5 and other rules but also can modulate thresholds according to specific requirements, providing more flexibility for screening drug candidates. Besides physicochemical properties, drug-likeness is also related to chemical absorption, distribution, metabolism, excretion, and toxicity (ADMET) properties. Recently, Guan et al. developed the ADMET-score to evaluate the drug-likeness of compounds based on 18 weighted ADMET properties [5]. The ADMET-score was able to distinguish withdrawn drugs from approved drugs with statistically significant accuracy.

Accurate prediction of drug-likeness is more complex than a simple counting of molecular properties. To improve the accuracy of drug-likeness prediction, researchers have adopted machine learning algorithms based on various molecular descriptors that enable more concrete representations of molecular information than basic chemical properties. Examples of these molecular descriptors include extended atom types [6], Ghose–Crippen fragment descriptors [7], molecular operating environment physicochemical descriptors [7], topological pharmacophore descriptors [7], ECFP4 fingerprints [8], LCFP6 fingerprints [9], and their combinations [7]. These descriptors, combined with various machine learning methods including support-vector machines [7,8,10], artificial neural networks [7], decision trees [6], naïve Bayesian classifiers [11], and recursive partitioning [11], have increased the accuracy of methods to discriminate drug-like and non-drug-like compounds to about 90%. In recent years, deep learning as a powerful machine learning method has been applied for drug-likeness prediction. Hu et al. proposed a deep autoencoder model to classify drug-like and non-drug-like compounds based on the synthetic minority oversampling technique (SMOTE), which achieved 96% accuracy [12]. Seyed et al. proposed a deep belief model based on a combination of fingerprints including MACCS, PubChem fingerprints, and ECFP4 [13], which yielded similar accuracy. Beker et al. combined different deep learning models using a Bayesian neural network and then chose the predictions of drug-like molecules with the lowest variance. According to their findings, the combination of graph convolution neural network (GCNN) and autoencoder yielded the highest accuracy of 93% [14]. Lee et al. generated a drug-likeness score function using unsupervised learning, which can predict drug-likeness with a continuous value rather than a rigid cut-off [15]. Their model also obtained over 90% accuracy in classifying drugs and non-drug molecules.

The aforementioned drug-likeness prediction models are typically trained using approved drugs (or a combination of approved drugs and candidate drugs in clinical trials) as positive samples and purchasable compounds as negative samples. However, there are situations where we are interested in determining the likelihood that a compound that is initially identified as being drug-like will be successful in the in vivo testing stage (referred to here as ‘in vivo ability’). Similarly, we may wish to forecast whether a compound in the in vivo testing stage will become an investigational new drug (referred to here as ‘IND ability’), or whether an investigational new drug will ultimately be approved to become a marketed drug (referred to here as ‘market approvability’). Failure in drug development can occur in various stages. For example, the clinical failure rate from phase I clinical trials to the successful launching of a drug on the market is over 90% [16]. The subdivisional drug-likeness prediction including market approvability prediction can therefore be useful for boosting success rates in clinical trials and other stages.

**Figure 1:**
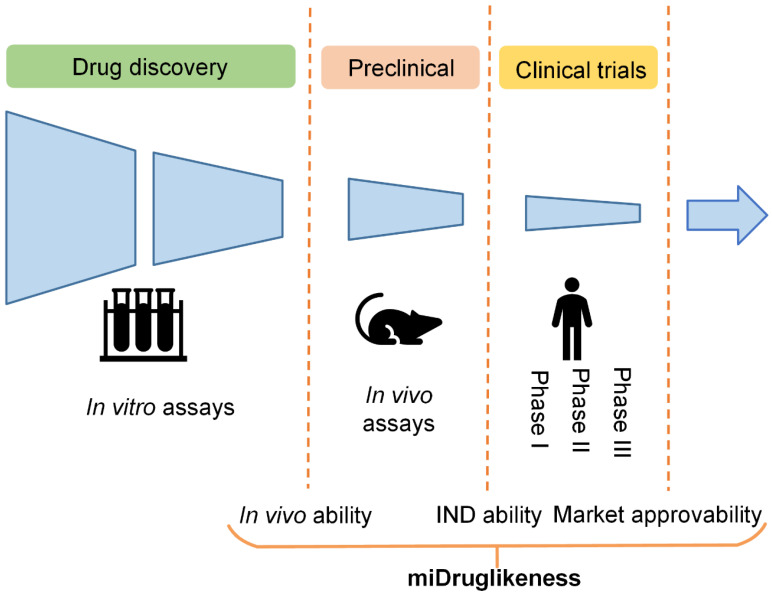
Drug-likeness prediction by stages.

In this study, we developed a miDruglikeness system including three subdivisional drug-likeness prediction models to deal with the abovementioned problems (Figure 1). The in vivo ability, IND ability, and market approvability models were respectively built using graph neural networks (GNNs). To enhance the predictive ability of these models, we combined active learning with ensemble learning. The miDruglikeness system showed satisfying performance in internal test datasets and self-collected external test datasets. The performance of miDruglikeness in the virtual screening test was significantly better than QED and state-of-the-art protein-ligand scoring-based methods. Besides, we also employed Shapley Additive exPlanation (SHAP) [17] to interpret these models and built a webserver of miDruglikeness for public usage.

## 2. Materials and Methods

### 2.1. Data Collection

All training datasets were obtained from the ZINC15 subsets, including the “world” subset, the “investigational only” subset, the “in trials” subset, the “in-vivo-only” subset, the “in-vivo” subset, and the “in-stock” subset [18]. Detailed information about these datasets is listed in Table 1. Due to the difficulty in gathering the negative samples in our tasks, we assumed that the compounds not entering the next stages in the time of data collection as negative samples because of the high failure rate in the drug development pipeline. For the in vivo ability models, the positive dataset was extracted from the “in-vivo” subset, and the negative dataset was extracted from the “in-stock” subset. For the IND ability models, the positive and negative datasets were constructed from the “in-trials” subset and the “in-vivo-only” subset, respectively. For the market approvability models, the positive dataset was extracted from the “world” subset and the negative dataset was extracted from the “investigational-only” subset. Compounds that appeared in both the positive dataset and the negative dataset were removed from the negative dataset. All datasets were “cleaned” in preprocessing steps using KNIME, an open-source platform that provides functionality and tools for data processing and analysis (see the preprocess workflow in Table 2). The market approvability dataset had the least amount of data, and all datasets were imbalanced (minor class samples/major class samples ratio). Detailed information on the final used datasets is shown in Table 3. Each dataset was randomly split into a 9(training):1(internal test) proportion. The training sets were randomly split five times into training sets and validation sets in 4:1 proportion for 5-fold cross-validation. The histograms of basic physicochemical properties of training sets are shown in Figures S1–S3. It is challenging to make a classification in the above three tasks based on physicochemical properties. 

To further evaluate the models’ reliability, we also construct the external test sets from reports and the public database. For in vivo ability prediction task, we combined self-collected compounds entering in vivo testing stage in recent years and the investigational group, the approved group in the DrugBank database [19] as the external test set, named in vivo compounds set. For the IND ability prediction task, we combined self-collected compounds entering clinical trials in recent years and the investigational group, the approved group in the DrugBank database [19] as the external test set, named the investigational compounds set. For the market approvability prediction task, we collected FDA-approved drugs in recent years and approved group data in DrugBank, named approved drugs set. It is harder for collecting negative samples than positive samples because the failure information usually was not reported. We only collected dozens of compounds terminated in clinical trials from reports and drugs that failed in clinical trials for toxicity reasons from the ClinTox database [20], named clinical terminated compounds set, which can serve as the negative test set for market approvability prediction task. All external sets were cleaned by removing duplicate data (also without duplication with training data). The details of the four datasets are shown in Table 4. We also used the DEKOIS 2.0 dataset [21] to test the virtual screening ability of the in vivo ability model. DEKOIS 2.0 library contains 81 benchmark sets for a wide variety of different target classes. The duplicate data in the DEKOIS 2.0 dataset with the training set were removed in the external testing. Detailed information on the DEKOIS2.0 dataset is shown in Supplementary Table S1.

### 2.2. Graph Neural Network

We constructed Direct Message Passing Neural Networks (D-MPNNs) for miDruglikeness model construction. DMPNNs have been used successfully for molecular prediction [22]. The DMPNN has two phases, a message phase, and a readout phase. In the message phase, information is transmitted through the graph architecture to the hidden states. The message update equation is:

(1) $$m_{vw}^{t+1}=\underset{k\in \left\{N\left(v\right)\backslash w\right\}}{\sum }M_{t}\left(x_{v},\;x_{k},\;h_{kv}^{t}\right)$$

(2) $$h_{vw}^{t+1}=U_{t}\left(h_{vw}^{t},\;m_{vw}^{t+1}\right)\;$$

where $m_{vw}^{t}$ is the message associated with a bond (from atom v to atom w) in step *t*, and $h_{vw}^{t}$ is the hidden state of bonds (from atom v to atom w) in step *t*. $M_{t}$ and $U_{t}$ are defined as follows:

(3) $$M_{t}\left(x_{v},\;x_{w},\;h_{vw}^{t}\right)=h_{vw}^{t}$$

(4) $$U_{t}\left(h_{vw}^{t},\;m_{vw}^{t+1\;}\right)=\tau \left(h_{vw}^{0},\;W_{m},m_{vw}^{t+1}\right)\;$$

where $W_{m}\in {\mathbb{R}}^{h\times h}$ is a learned matrix with hidden size h.

In the readout phase, the representation of all hidden states is aggerated into a final representation for prediction tasks. All final hidden states $h_{v}$ of atom can be obtained by

(5) $$m_{v}=\underset{w\in N\left(v\right)}{\sum }h_{vw}^{T}$$

(6) $$h_{v}=\tau \left(W_{a}cat\left(x_{v},m_{v}\right)\right)$$

Then we can get the prediction $\hat{y}$ through a feed-forward neural network f as follows

(7) $$h=\underset{v\in G}{\sum }h_{v}$$

(8) $$\hat{y}=f\left(h\right)$$

**Figure 2:**
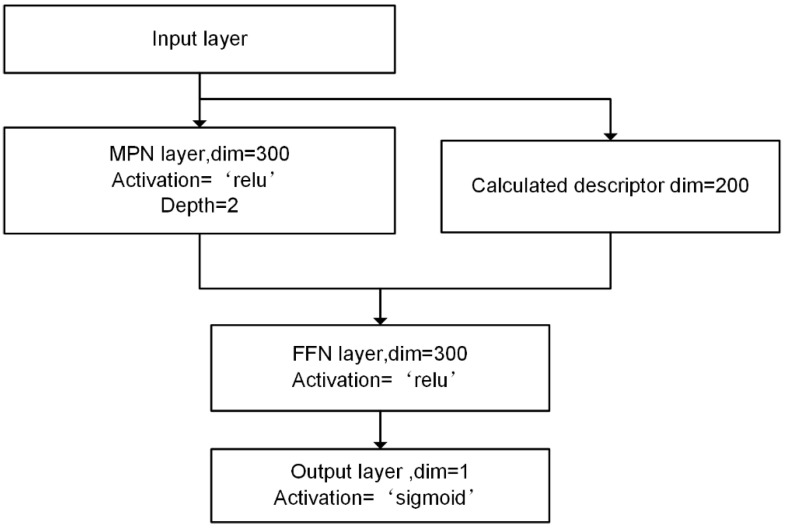
The architecture of D-MPNN models for miDruglikeness.

The D-MPNN network was implemented using a Python package, ChemProp. The hyperparameters were optimized by grid search. The three models adopted the same network architecture but different learning rates including 1e-3, 1e-3, and 5e-4, respectively. The depth of MPN (message passing neural network) layers and feedforward neural network layer was 2. The hidden size was 300 and the batch size was 500. During the training process, early stopping was used to avoid overfitting. Besides, we calculated the 200 molecular features through RDkit to enhance model performance. The architecture of miDruglikeness models is shown in Figure 2. 

### 2.3. Active Ensemble Learning

Active learning is a machine learning strategy for situations where unlabeled data are abundant or easily obtained but data labeling is difficult, time-consuming, or expensive [23], which makes it well suited for drug discovery. During the process of active learning, the model can select the most informative samples from an unlabeled dataset, and the selected data can be labeled by experiments. By using iterative selection with an active learning query strategy, the model can get satisfactory performance with a relatively small amount of training data, which can save downstream labeling and experimental costs. 

**Figure 3:**
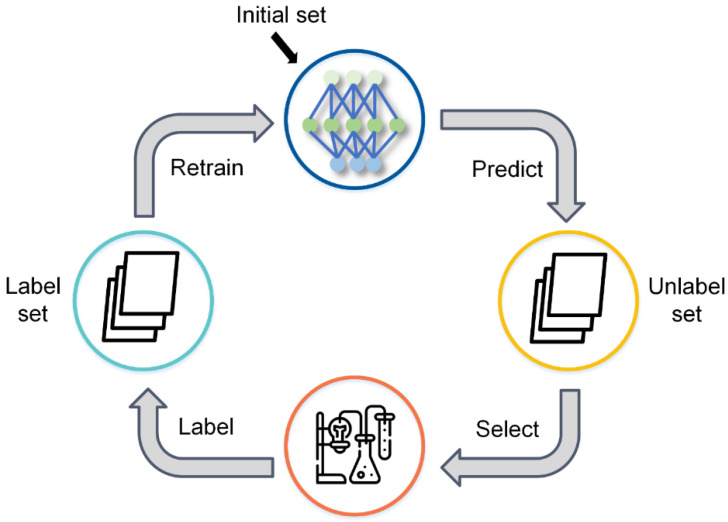
The schematic diagram of active learning.

We constructed a pool-based active learning trainer based on ALiPy [24], an open-source active learning framework. Labels of the entire sample pool are stored in a cache and can be queried by the trainer using a query function. Figure 3 depicts the active learning training process. First, a small percentage of training samples (5% in our task) are labeled to initialize the model. Then, the remaining unlabeled samples are predicted by the trained model and checked with the query function to get the score for selection. According to the selection score, the trainer selects a batch of samples and adds them to the training dataset with their corresponding labels. The model is then retrained using the new training dataset. After the model has been trained, the next iteration begins. To reduce the number of iterations and speed up the training, the batch size of the query for each iteration is not the same but rather is denser at the beginning and sparser at the end. 

We adopted uncertainty sampling for selection [23] because the most uncertain samples are considered to have the most information. For the classification tasks, we wanted to focus on the most uncertain samples at the margin. The score function calculates the uncertainty of the samples and is defined as:

(9) $$x_{uncertainty}=1-P\left(\hat{y}|x\right)$$

(10) $$\;\hat{y}=\mathrm{argmax}\;P\left(y_{i}|x\right)\;$$

where x is the specific sample, $x_{uncertainty}$ is the uncertainty of the sample, and $P\left(y_{i}|x\right)$ is the predicted probability of *i*-th class for the sample.

**Figure 4:**
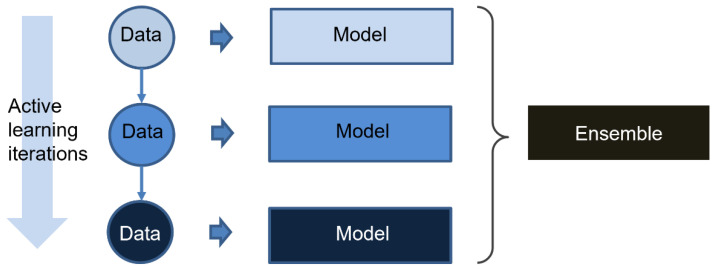
The schematic diagram of active ensemble learning strategy.

Ensemble learning is a popular machine learning technique that combines several base learners to produce one optimal predictive model. It is generally believed that base learners used for ensemble learning should have some diversity [25]. The diversity can be developed at the data level (boosting or bagging) as well as at the algorithm level (stacking). Because the training dataset in each iteration of active learning is expanded by new data that are ambiguous for the model in the last iteration, the models in each iteration of active learning are actually trained by different data. Thus, the models in the active learning process are used as base learners for ensemble learning in our study. The workflow of the active learning strategy is shown in Figure 4. The final output is as follows:

(11) $$y=\frac{1}{N}\overset{N}{\underset{n=1}{\sum }}y_{n}\;$$

where y is the final output, and $y_{n}$ is the prediction of base learners.

For baseline control, we trained a D-MPNN iteratively with random picking (passive learning) and combined the passive learning models in the same way as the active learning models, called passive ensemble learning. 

### 2.4. Model Evaluation Metrics

Our models were evaluated by three metrics: accuracy (ACC), Matthews correlation coefficient (MCC), and F1-score. Because ACC is sometimes not a good metric for imbalanced datasets, we further adopted both MCC and F1-score, which are better suited for imbalanced data. For virtual screening testing, the AUC (area under curve) of receiver operating characteristic (ROC) and enrichment factor (${\mathrm{EF}}_{\gamma }$) were used.

(12) $$\mathrm{ACC}=\frac{\mathrm{TP}+\mathrm{TN}}{\mathrm{TP}+\mathrm{TN}+\mathrm{FP}+\mathrm{FN}}\;$$

(13) $$\mathrm{MCC}=\frac{\mathrm{TP}\times \mathrm{TN}-\mathrm{FP}\times \mathrm{FN}}{\sqrt{\left(\mathrm{TP}+\mathrm{FP}\right)\left(\mathrm{TP}+\mathrm{FN}\right)\left(\mathrm{TN}+\mathrm{FP}\right)\left(\mathrm{TN}+\mathrm{FN}\right)}}\;$$

(14) $$\mathrm{Precision}=\frac{\mathrm{TP}}{\mathrm{TP}+\mathrm{FP}}$$

(15) $$\mathrm{Recall}\;=\frac{\mathrm{TP}}{\mathrm{TP}+\mathrm{FN}}\;$$

(16) $$F1\;\mathrm{score}=2\times \frac{\mathrm{Precision}\times \mathrm{Recall}}{\mathrm{Precision}+\mathrm{Recall}}$$

(17) $${\mathrm{EF}}_{\gamma \;}=\frac{{\mathrm{NTB}}_{\gamma }}{{\mathrm{NTB}}_{\mathrm{total}}\times \gamma }\;$$

where TP is the number of true positive samples, TN is the number of true negative samples, FP is the number of false positive samples, and FN is the number of false negative samples. ${\mathrm{NTB}}_{\gamma }$ is the number of the true active samples among the top predictions with cutoff γ setting. ${\mathrm{NTB}}_{\mathrm{total}}$ is the number of true active samples in the total prediction list.

## 3. Results

### 3.1. Performance of miDruglikenss System on the Internal Tests

**Figure 5:**
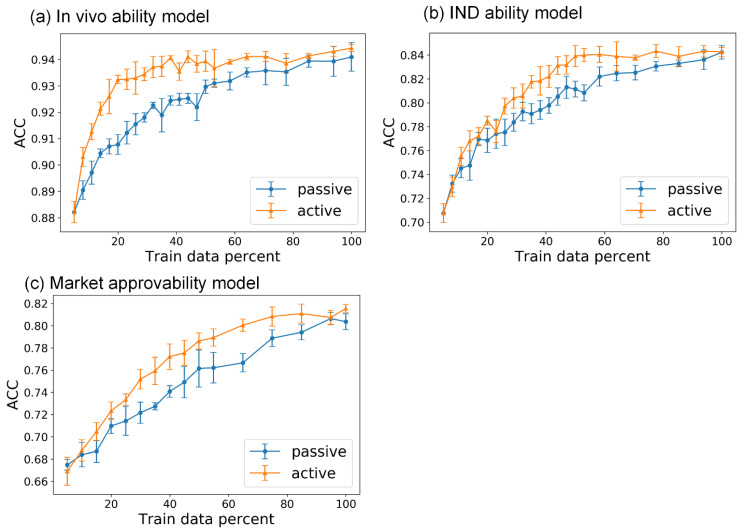
The ACC of models in the active learning process on test sets versus the percentage of training data used. (**a**) In vivo ability model; (**b**) IND ability model; (**c**) market approvability model. The blue lines represent the performance of passive learning models, whereas the orange lines represent the performance of active learning models, and the error bar is the standard deviation of testing results in five-fold training.

For active learning, we randomly selected 5% of the data in the training set as the initial labeled data pool and left the remaining data to construct the unlabeled data pool. After selection in each iteration of active learning, the selected data of the unlabeled data pool were directly added to the labeled data pool. We used passive learning, in which the data selection criteria are random, as a baseline control. For in vivo ability prediction, the active learning models quickly reached maximum performance, whereas the passive learning models did not reach maximum performance until the end of the iteration (Figure 5a). All of the metrics used to evaluate the performance of the models exhibited similar tendencies. The active learning models were able to achieve maximum performance with about 40% of the whole training dataset, but the passive learning models required 100% of the training data (the results of MCC and F1 are shown in Figures S4 and S5). Similarly, for the models predicting IND ability and market approvability, active learning models achieved maximum performance with about 50% and 60% of the whole training datasets, respectively, whereas passive learning models required all of the training data (Figure 5b,c). These results showed that the models had sufficient predictive capacity in the plateau of the active learning process to generate meaningful predictions for effective drug development. 

**Figure 6:**
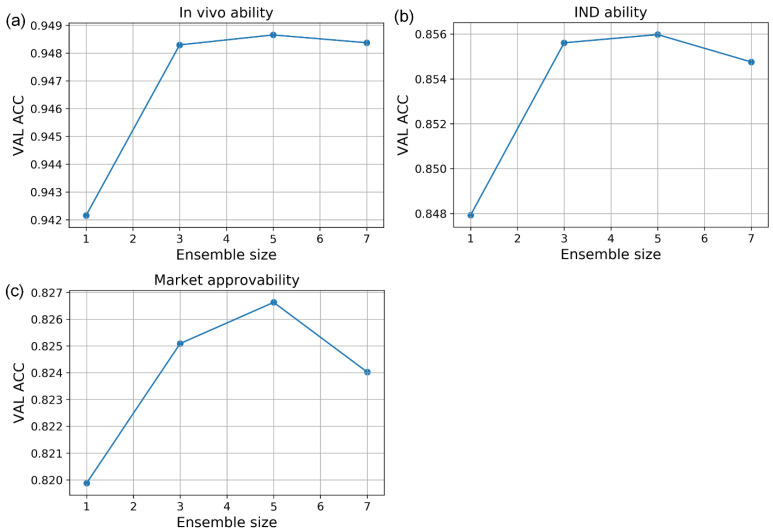
The ACC vs. ensemble size curve of active learning strategies on validation sets. (**a**) In vivo ability model; (**b**) IND ability model; (**c**) market approvability model.

To further improve performance, we combined these models using ensemble learning. We integrated the models of the final few iterations in the performance plateau of Figure 5 using an averaging sum method (Equation (11)). We did not include the models in the early iterations because of their inferior prediction accuracy, which may hurt the performance of ensemble learning. For all three prediction tasks, we experimented with different ensemble sizes and chose the size that gave the best overall performance in the validation set. The three models obtained the best results when using the last five models in the active learning iterations for ensemble learning (Figure 6). 

We compared the results of the active ensemble learning strategy with a powerful traditional machine learning method, XGBoost [26], and other ensemble strategies (Table 5). The XGBoost was trained with 200 molecular features (including physical properties and fragments descriptors, etc.) through RDkit and the hyperparameters were optimized by grid-based random search. The normal learning model was a D-MPNN trained on the whole training dataset, which showed better performance than XGBoost in in vivo ability prediction task and market approvability prediction task. Active ensemble learning further improved the D-MPNN model performances across all metrics. MCC values grew by an average of 3% with active ensemble learning compared with normal learning, while the other performance metrics increased by 1–2%. We also contrasted the active ensemble learning strategy with another two ensemble strategies. The normal ensemble learning strategy integrated models trained by normal learning (without active learning) on whole training datasets, whereas the passive ensemble learning strategy integrated models trained by passive learning. The ensemble sizes for normal ensemble learning and passive ensemble learning were the same as those used for active ensemble learning. Active ensemble learning models achieved the best or comparable performance among all the strategies (Table 5). Besides, active ensemble strategies also obtained the best performance in a balanced test (Figure S6). Finally, we adopted active ensemble learning models for the miDruglikeness system. The in vivo ability prediction model in miDruglikeness showed the highest performance in all three models, which attained state-of-the-art accuracy (above 94%) and MCC value (84.7%). The IND ability prediction model and market approvability prediction model also achieved satisfactory accuracy above 80% with smaller training datasets (Table 6).

To further assess the efficacy of active ensemble learning, we evaluated our active ensemble learning strategy on tasks using some ChEMBL data [27] and found that active ensemble learning still achieved the best performance in all strategies used (see Supplementary Table S3), which implies that our active ensemble learning strategy is a general performance-enhancing strategy other than the drug-likeness task. 

**Figure 7:**
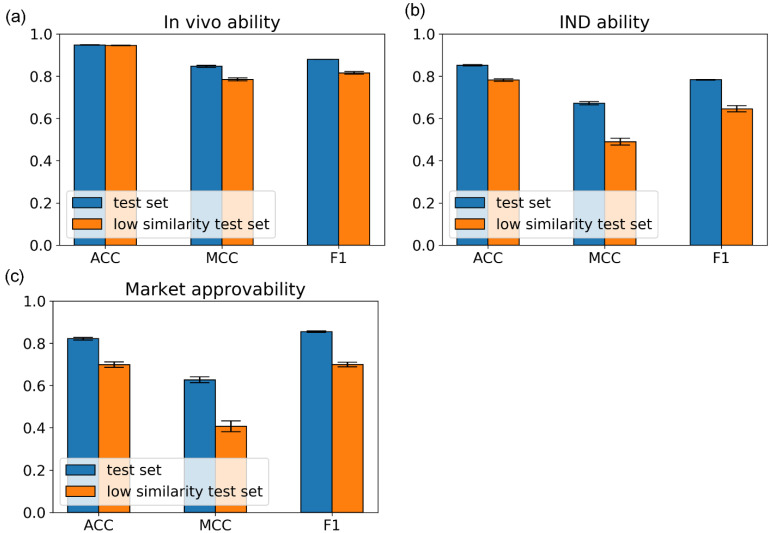
The performance of miDruglikeness models on the internal test set and the low similarity test set, the error bar is the standard deviation of testing results in five-fold training. (**a**) In vivo ability model; (**b**) IND ability model; (**c**) market approvability model.

Because drugs have some analogs of previous drugs. To evaluate the performance of miDruglikeness models on the dissimilar data, we excluded test set molecules with similarity above 0.85 to the molecules in the training set to construct a stricter test set, i.e., the low similarity test set. The similarity was calculated using Tanimoto coefficient similarity on the ECFP4 fingerprints. As shown in Figure 7, the ACC value of the in vivo ability model still had a high value of 94.6% and other metrics also with a high level of approximately 80%. The ACC values of the IND ability model and the market approvability model are above 70% but MCC and F1-score decreased more, which was mainly affected by the data imbalance. The results illustrated that miDruglikeness models had good generalizability for dissimilar compounds. 

### 3.2. The Performance of miDruglikenss System on the External Tests

**Figure 8:**
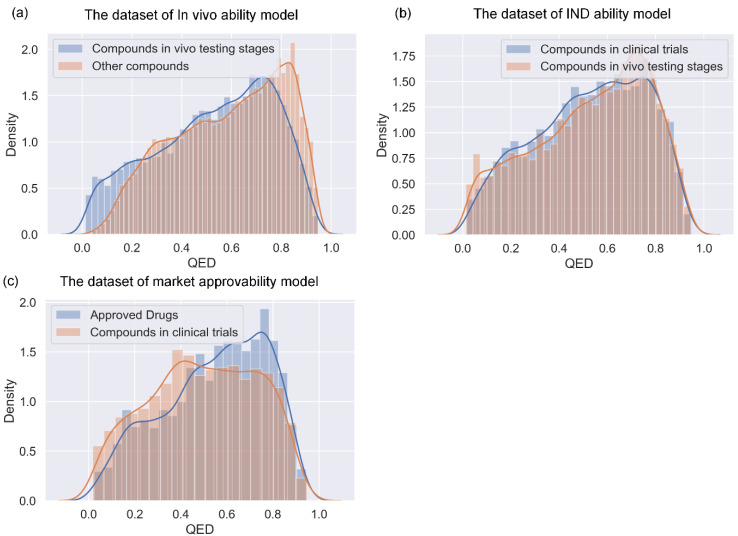
The QED distribution of training datasets of miDruglikeness. (**a**) In vivo ability model; (**b**) IND ability model; (**c**) market approvability model.

We further tested miDruglikeness models with an evaluation of the external sets and make a comparison to QED. Because all external sets only have one class, ACC is the only evaluation metric. As indicated in Table 7, the performance of the miDruglikeness models was significantly better than that of QED. The ACC of QED of all external sets was about 50%, close to random predictions. miDruglikeness demonstrated the best performance in in vivo ability prediction task (in vivo compounds set), which is consistent with our internal evaluation. Moreover, miDruglikeness obtained over 70% accuracy on the clinical terminated compounds set, showing a good predictive ability for filtering failed compounds in clinical trials, which is useful for saving costs in clinical trials. In the external test to distinguish the investigational compounds and drugs from ChEMBL, miDruglikeness still obtained a high accuracy of 72.8% (Table S5). By analyzing the QED distribution of training datasets in miDruglikeness tasks (Figure 8), we discovered that QED is not discriminating in subdivisional drug-likeness prediction tasks. 

### 3.3. In Vivo Ability Model for Virtual Screening

Given that in vivo ability prediction task is closely related to the compound bioactivity, we evaluated the in vivo ability prediction model on DEKOIS 2.0 data set. Because the in vivo ability model was a ligand-based model, we employed a widely used docking program Autodock Vina (Vina) [17] as a baseline of comparison, which also did not use a target-specific training set. In addition, we also compared our results with a recently published target-based scoring function, DyScore [28]. For enrich factor (EF) calculation, we utilized the predicted probability of in vivo ability as the ranking score. The in vivo ability prediction model showed very good performance in virtual screening tasks and significantly performed better than Vina and DyScore, though no protein targets information nor interaction information is considered in our model (Table 8). The detailed virtual screening test results for each target are included in Supplementary Table S6.

### 3.4. Model Interpretation

**Figure 9:**
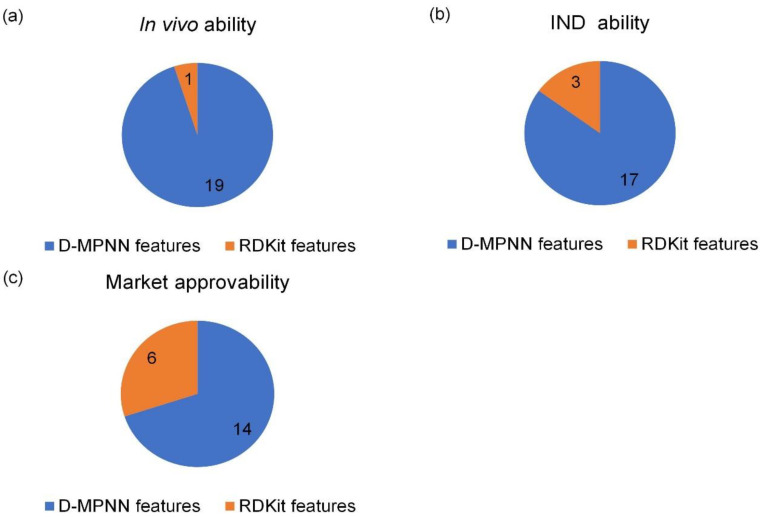
The top 20 important features of miDruglikeness models. (**a**) In vivo ability model; (**b**) IND ability model; (**c**) market approvability model.

We analyzed the importance of D-MPNN features and RDkit descriptors we adopted using SHAP (see details in Supplementary). SHAP is a game theory method used to explain the output of a machine learning model with Shapley values. The importance of features of the miDruglikeness models can be used to determine how features affect the prediction of models. D-MPNN features are the hidden vectors of molecules through message-passing neural network layers in the D-MPNN model. They represent the features extracted by the molecular graph with D-MPNN. Since the D-MPNN features are latent and information-rich vectors, they are difficult to further interpret. But we observed that D-MPNN features played a major role in decision making, while RDkit descriptors played an auxiliary role. The better the model was trained, the more D-MPNN features were prominent. The in vivo ability prediction model only had one RDkit feature in the top 20 important features, while the market approvability model had 6 RDkit features (Figure 9). The description of these important RDkit features is shown in Table 9. 

### 3.5. The miDruglikeness Webserver

We developed the miDruglikeness webserver for convenient use of our tool. The users can simply submit compounds in SMILES (simplified molecular input line entry system) format or submit a text file containing compounds in SMILES strings to obtain miDruglikeness predictions. A CSV file containing the predicted labels as well as the predicted probability of three models will be returned to the users. The webserver is freely available at http://www.pkumdl.cn:8000/midruglikeness/ (accessed on 1 November 2022). 

## 4. Discussion

In this work, we first proposed and implemented three subdivisional models to individually predict the possibility of a compound entering the in vivo testing, clinical trials, and market approval stages. By making drug-likeness predictions in this subtle manner, we can predict the probability of a compound entering the next stage of drug discovery and development. Our testing results demonstrated the robustness and the generalization ability of miDruglikeness models. Although docking approaches based on target information are popular for virtual screening, their performance is inadequate. Our in vivo ability model showed a very good performance for virtual screening. Since in vivo ability model is target independent, it would serve as an orthogonal complementary to target-based virtual screening methods and is anticipated to considerably increase the success rate of virtual screening. Many researchers utilize QED as a filter in virtual screening. However, we discovered that our in vivo ability model worked far better than QED. Thus, we highly recommend that users can employ in vivo ability as a strong filter in their virtual screening campaigns.

The miDruglikeness models are mainly based on the structural information of compounds. Nevertheless, drug approval is a complicated process that is also affected by biological and commercial factors. Thus, the IND ability prediction task and market approvability prediction are more challenging than in vivo ability prediction task. To further improve the predictive performance of the IND ability model and the market approvability model, additional information such as biological data from in vivo assays and clinical trials should also be considered. 

We developed the active ensemble learning method for miDruglikeness models in this study. Additionally, active ensemble learning is a general strategy for machine learning models with uncertainty and can be used for a variety of different tasks, such as the prediction of bioactivities. Active ensemble learning can combinedly be used with other methods such as XGBoost and DNN. From Figure S7, we also discovered that active learning could maintain data balance at a certain range in the data selection. It could be applied for scenarios when sampling imbalanced data.

## 5. Conclusions

We have developed a miDruglikeness system consisting of three models to predict in vivo ability, IND ability, and market approvability to deal with scenarios corresponding to the different stages of drug development. Active ensemble learning as a general strategy was developed and applied to further enhance the miDruglikeness models. These models are demonstrated to have satisfactory performance on internal test datasets, four external test datasets, and the virtual screening tests. We believe the miDruglikeness models offer better indexes than QED for rapid evaluation of a large amount of molecular data such as molecules generated by deep generative networks. We also built the miDruglikeness webserver to facilitate usage.

## Figures and Tables

**Table 1:** Descriptions of the ZINC 15 subsets used in this work.

Dataset Name	Description
world	Approved drugs in major jurisdictions, including the FDA.
investigational-only	Compounds in clinical trials, not approved or used as drugs.
in-trials	Compounds that have been investigated, including drugs.
in-vivo-only	Substances tested in animals but not in humans.
in-vivo	Substances tested in animals including humans.
in-stock	Compounds purchased directly from a manufacturer, already made, sitting on a shelf, ready to ship.

**Table 2:** The data “cleaning” preprocess workflow.

Step	Action
Organic filter	Inorganic molecules are removed.
Element filter	Molecules containing elements other than C, H, O, N, P, S, Cl, Br, I, and F are removed.
Connectivity	Fragments except for the biggest from unconnected molecules are removed.
Standardizer	Directly bonded zwitterions are converted to the neutral representation. The charges on a molecule are set to a standard form.
Aromatizer	The molecules are converted into an aromatic form.
Canonicalize	The molecules are represented in canonical SMILES format.
Duplicate filter	Duplicate molecules are removed.

**Table 3:** Dataset information after data “cleaning”.

Task	Data Subset in ZINC15 (Positive or Negative in Datasets)	Compound Number	Imbalance Ratio
In vivo ability	in-vivo (+)	27329	3.65
	in-stock (−)	99812	
IND ability	in-trials (+)	9386	1.91
	in-vivo-only (−)	17943	
Market approvability	world (+)	5706	1.55
	investigational-only (−)	3680	

**Table 4:** The summary of the external test sets.

Name	Description	Source	Size	Total Size (after Removing Duplicates)
In vivo compounds set	Compounds successfully entering into in vivo test stages.	Self-collect	112	1369
		DrugBank	1269	
Investigational compounds set	Compounds successfully entering into in clinical trials.	Self-collect	103	1326
		DrugBank	1270	
Approved drugs set	Compounds successfully being market-approved.	Self-collect	70	408
		DrugBank	395	
Clinical terminated compounds set	Compounds terminated in clinical trials.	Self-collect	21	24
		ClinTox	14	

**Table 5:** The performance of active learning methods and other different methods on internal test sets.

Task	Methods	ACC	MCC	F1
In vivo ability prediction	XGBoost	0.932 ± 0.001	0.799 ± 0.003	0.842 ± 0.002
	Normal learning	0.941 ± 0.003	0.824 ± 0.010	0.860 ± 0.000
	Passive ensemble	0.945 ± 0.003	0.838 ± 0.010	0.873 ± 0.000
	Normal ensemble	0.945 ± 0.003	0.838 ± 0.009	0.872 ± 0.000
	Active ensemble	**0.948 ± 0.001**	**0.847 ± 0.005**	**0.880 ± 0.000**
IND ability prediction	XGBoost	0.849 ± 0.003	0.661 ± 0.008	0.771 ± 0.005
	Normal learning	0.832 ± 0.015	0.640 ± 0.019	0.770 ± 0.001
	Passive ensemble	0.846 ± 0.004	0.657 ± 0.007	0.771 ± 0.002
	Normal ensemble	0.851 ± 0.003	0.670 ± 0.005	**0.784 ± 0.002**
	Active ensemble	**0.852 ± 0.003**	**0.672 ± 0.008**	0.783 ± 0.002
Market approvability prediction	XGBoost	0.809 ± 0.004	0.599 ± 0.009	0.847 ± 0.003
	Normal learning	0.811 ± 0.008	0.606 ± 0.016	0.844 ± 0.005
	Passive ensemble	0.808 ± 0.007	0.597 ± 0.015	0.843 ± 0.004
	Normal ensemble	0.819 ± 0.004	0.621 ± 0.009	0.854 ± 0.003
	Active ensemble	**0.822 ± 0.006**	**0.627 ± 0.014**	**0.855 ± 0.003**

The bold numbers are the best results, the standard deviations are from the five-fold training/test.

**Table 6:** The performance of miDruglikeness models on the validation sets and internal test sets.

		ACC	MCC	F1
In vivo ability model	Validation	0.949 ± 0.002	0.846 ± 0.004	0.878 ± 0.004
	Test	0.948 ± 0.001	0.847 ± 0.005	0.880 ± 0.004
IND ability model	Validation	0.856 ± 0.002	0.675 ± 0.006	0.782 ± 0.007
	Test	0.852 ± 0.003	0.672 ± 0.008	0.783 ± 0.008
Market approvability model	Validation	0.827 ± 0.011	0.633 ± 0.023	0.860 ± 0.010
	Test	0.822 ± 0.006	0.627 ± 0.014	0.855 ± 0.005

The standard deviations are from the five-fold training/test.

**Table 7:** The predicting ACC values of miDruglikeness models and QED on the external sets.

	In Vivo Compounds Set	Investigational Compounds Set	Approved Drugs Set	Clinical Terminated Compounds Set
QED	0.515	0.494	0.529	0.500
miDruglikeness	0.773	0.620	0.662	0.708

**Table 8:** The virtual screening performance of the in vivo ability model, DyScore and Vina on the DEKOIS 2.0 dataset *.

	AUC	EF_1%_	EF_2%_	EF_5%_	EF_10%_
Vina	0.631	4.8	4.0	2.8	2.3
DyScore	0.702	7.5	6.2	4.3	3.3
miDruglikeness	0.860	9.2	9.1	7.3	5.6

* Data for Vina and DyScore are from ref [28]. As 4 targets (A2A, HDAC2, PPAR-1, and PPARA) were not tested by DyScore, all the data are calculated without these 4 targets.

**Table 9:** Description of the RDkit features in the top 20 important features of miDruglikeness.

Tasks	Feature	Description
In vivo ability	Chi2n	Similar to Hall–Kier Chi2v, but uses nVal instead of valence.
IND ability	NumAliphaticRings	The number of aliphatic (containing at least one non-aromatic bond) rings for a molecule.
	MolLogP	Wildman–Crippen LogP value.
	FractionCSP3	The fraction of C atoms that are sp^3^ hybridized.
Market approvability	Kappa2	Hall–Kier Kappa2 value.
	NumAromaticRings	The number of aromatic rings for a molecule.
	SlogP_VSA10	MOE logP VSA Descriptor 10 (0.40 ≤ x < 0.50).
	NumSaturartedRings	The number of saturated rings for a molecule.
	fr_bicyclic	Bicyclic.
	fr_AI_OH	Number of aliphatic hydroxyl groups.

## Data Availability

The code and data are provided at https://github.com/tytcc/miDruglikeness (accessed on 1 November 2022).

## Supplementary Materials

The following supporting information can be downloaded at: https://www.mdpi.com/article/10.3390/biom13010029/s1, Figure S1: The histograms of 6 basic physicochemical properties of in vivo ability training sets; Figure S2: The histograms of 6 basic physicochemical properties of IND ability training sets; Figure S3: The histograms of 6 basic physicochemical properties of market approvability training sets; Figure S4: The MCC of models in the active learning process on test sets versus the percentage of training data used; Figure S5: The F1 of models in the active learning process on test sets versus the percentage of training data used; Figure S6: The performance of different methods on balanced test sets; Figure S7: The balance ratio of training set in the active learning iterations; Table S1: Detailed information of DEKOIS2.0; Table S2: The detailed information balance test data; Table S3: The performance of different strategies on ChEMBL data; Table S4: The detailed information of investigational compounds and drugs from ChEMBL after removing duplicates and preprocessing; Table S5: The performance of market approvability model on ChEMBL data; Table S6: The virtual screening performance of in vivo ability model on DEKOIS 2.0; Table S7: The detailed information of bioactivity data.
