# Supplementary material for: miDruglikeness: Subdivisional Drug-Likeness Prediction Models Using Active Ensemble Learning Strategies
Source: Biomolecules. 2022 Dec 23;13(1):29. doi: 10.3390/biom13010029 (PMC9855665; doi:10.3390/biom13010029)
Supplement: Supplementary file 1 [file biomolecules-13-00029-s001.zip › biomolecules-2057493-supplementary.pdf]

Supplemental Information*:*

miDruglikeness: subdivisional drug-likeness prediction models using active ensemble learning strategies

Chenjing Cai[1]*, Haoyu Lin*[2]*, Hongyi, Wang*[2], *Youjun, Xu*[3]*, Qi Ouyang,*[1,4] *Luhua Lai,*[1,2,5] *Jianfeng Pei*[1,4]*

[1]Center for Quantitative Biology, Academy for Advanced Interdisciplinary Studies, Peking University, Beijing, 100871, China

[2]BNLMS, State Key Laboratory for Structural Chemistry of Unstable & Stable Species, College of Chemistry and Molecular Engineering, Peking University, Beijing, 100871, China

[3]Infinite Intelligence Pharma, Beijing 100083, China

[4]The State Key Laboratory for Artificial Microstructures and Mesoscopic Physics, School of Physics, Peking University, Beijing 100871, China

[5]Research Unit of Drug Design Method, Chinese Academy of Medical Sciences (2021RU014), Beijing 100871, China

*Correspondence: jfpei@pku.edu.cn (J.P.)

Interpreting the miDruglikeness models with SHAP:

Because SHAP cannot directly interpret the GNNs. We first used the trained D-MPNN models to calculate the D-MPNN features of datasets and then concatenated them with RDkit descriptors as input of the feed-forward network with the same config of miDruglikeness models. After training the feed-forward network, we can directly apply the GradientExplainer to get the importance score of D-MPNN features and RDkit features.

Balanced data test

To further test our model, we also built the balanced test data with down sampling to eliminate the effect of imbalance in the test data. The data information is shown in **Table S2**. The active ensemble learning still showed the best performance and obtained above 80% accuracy value in all three tasks. The results are shown in **Figure S6**. The active ensemble learning is a general strategy that can improve model's performance.

External test for investigational compounds and drugs in ChEMBL

We collected the "investigational" and "drug" compounds in ChEMBL. After removing duplicates with training sets and preprocessing, there are only 1802 compounds left. Thus we used it as an external test set to test our market approvability model. The market approvability model obtained a satisfying ACC value of 0.728. The datasets and results are shown in **Table S4** and **Table S5**.

Active ensemble learning for bioactivity prediction

We also tested active ensemble strategies on bioactivity prediction. We collected active compounds and inactive compounds for four targets from ChEMBL. The details of datasets are

showed in **Table S7**. And the active ensemble showed better performance than other ensemble strategies. The results are shown in **Table S3**.

The balance ratio in active learning iterations

We calculated the balance ratio of training data in the active learning iterations and passive learning iterations. The balance ratio is relatively high in the early stage of active learning iterations and the balance ratio decreased at the end stage because the left unlabeled pool is imbalanced (**Figure S7**). However, the balance ratio is still low in the passive learning iterations. This suggested that active learning can keep data balanced to some extent. The balance ratio equation is

$$Balance\ ratio = \frac{N_{minority\ class}}{N_{majority\ class}} \qquad (S1)$$

where $N_{minority\ class}$ is the number of molecules in minority class, $N_{majority\ class}$ is the number of molecules in majority class.

The figures and tables:

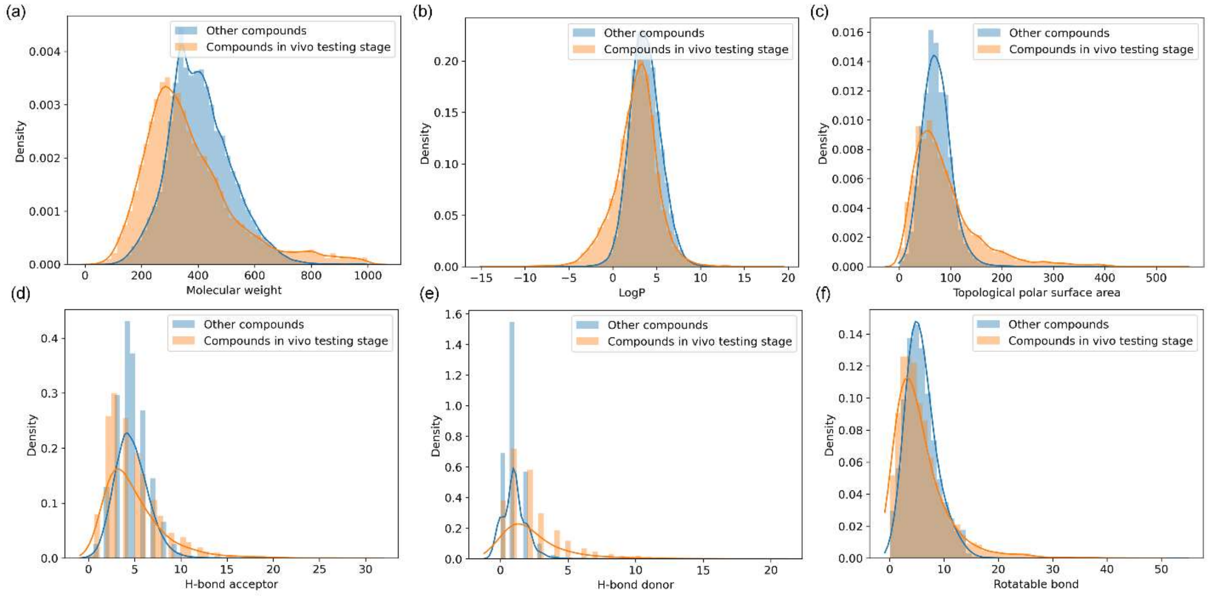

Figure S1. The histograms of 6 basic physicochemical properties of *in vivo* ability training sets. (a) Molecular weight (b) LogP (c) Topological polar surface area (d) H-bond acceptor (e) H-bond donor (d) Rotatable bonds

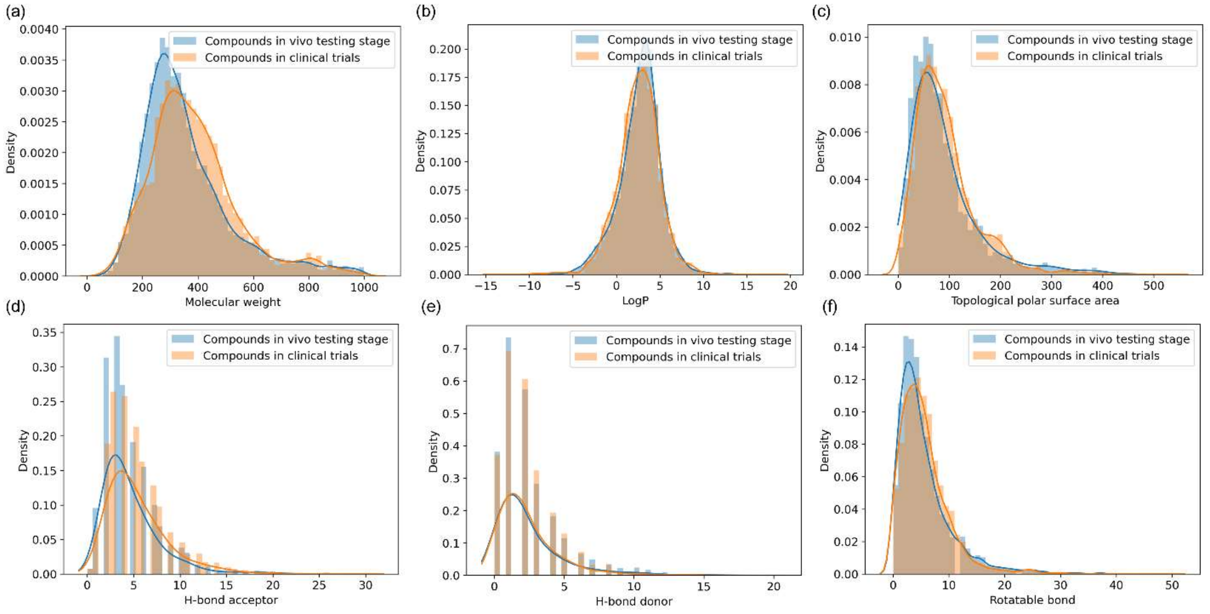

Figure S2. The histograms of 6 basic physicochemical properties of IND ability training sets. (a) Molecular weight (b) LogP (c) Topological polar surface area (d) H-bond acceptor (e) H-bond donor (d) Rotatable bonds

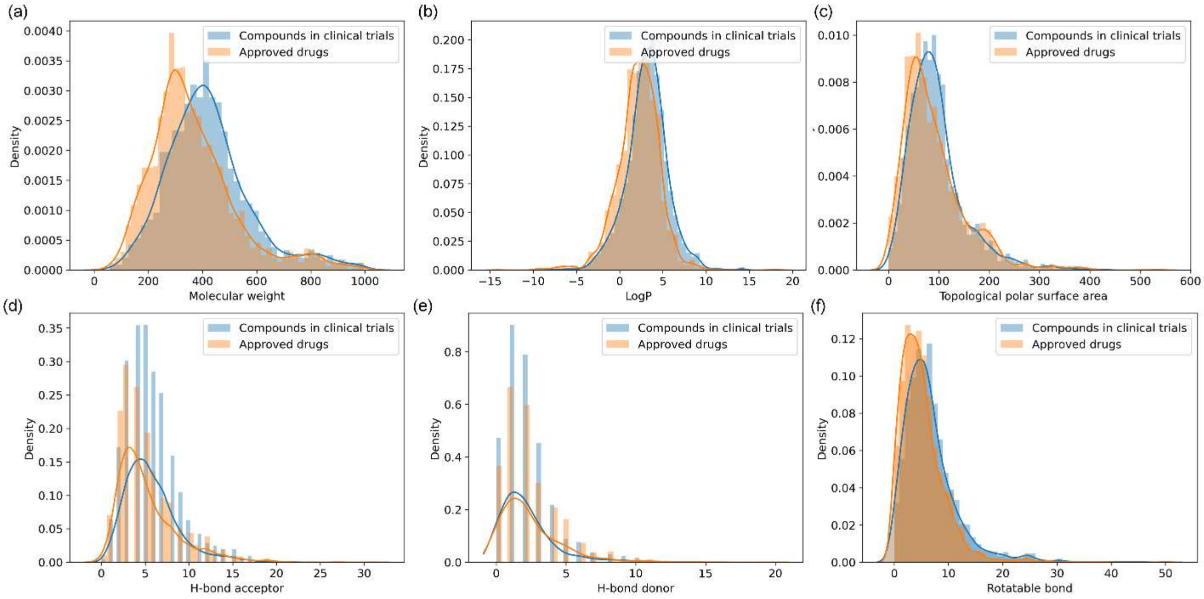

Figure S3. The histograms of 6 basic physicochemical properties of market approvability training sets. (a) Molecular weight (b) LogP (c) Topological polar surface area (d) H-bond acceptor (e) H-bond donor (d) Rotatable bonds

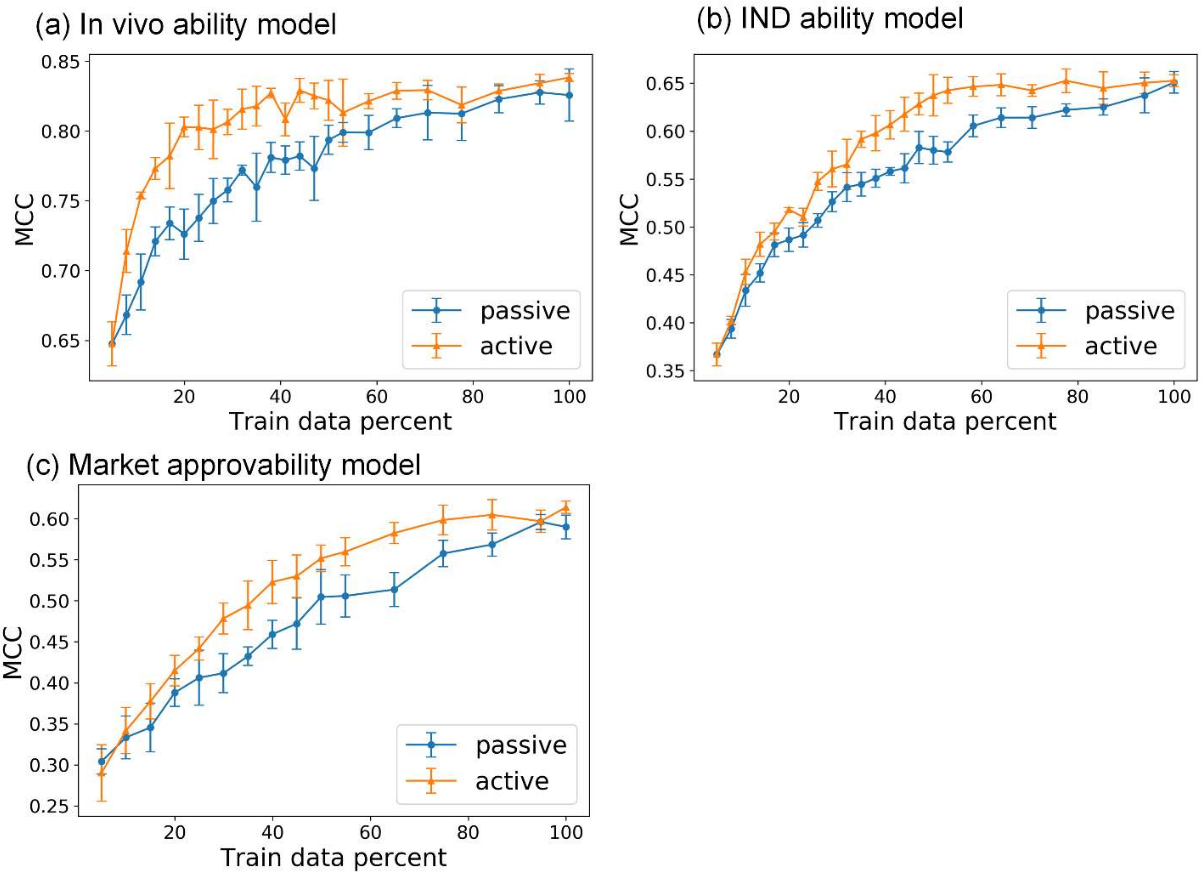

Figure S4. The MCC of models in the active learning process on test sets versus the percentage of training data used. (a) *In vivo* ability model (b) IND ability model (c) Market approvability model. The blue lines represent the performance of passive learning models, whereas the orange lines represent the performance of active learning models, and the error bar is the standard deviation of testing results in five-fold training.

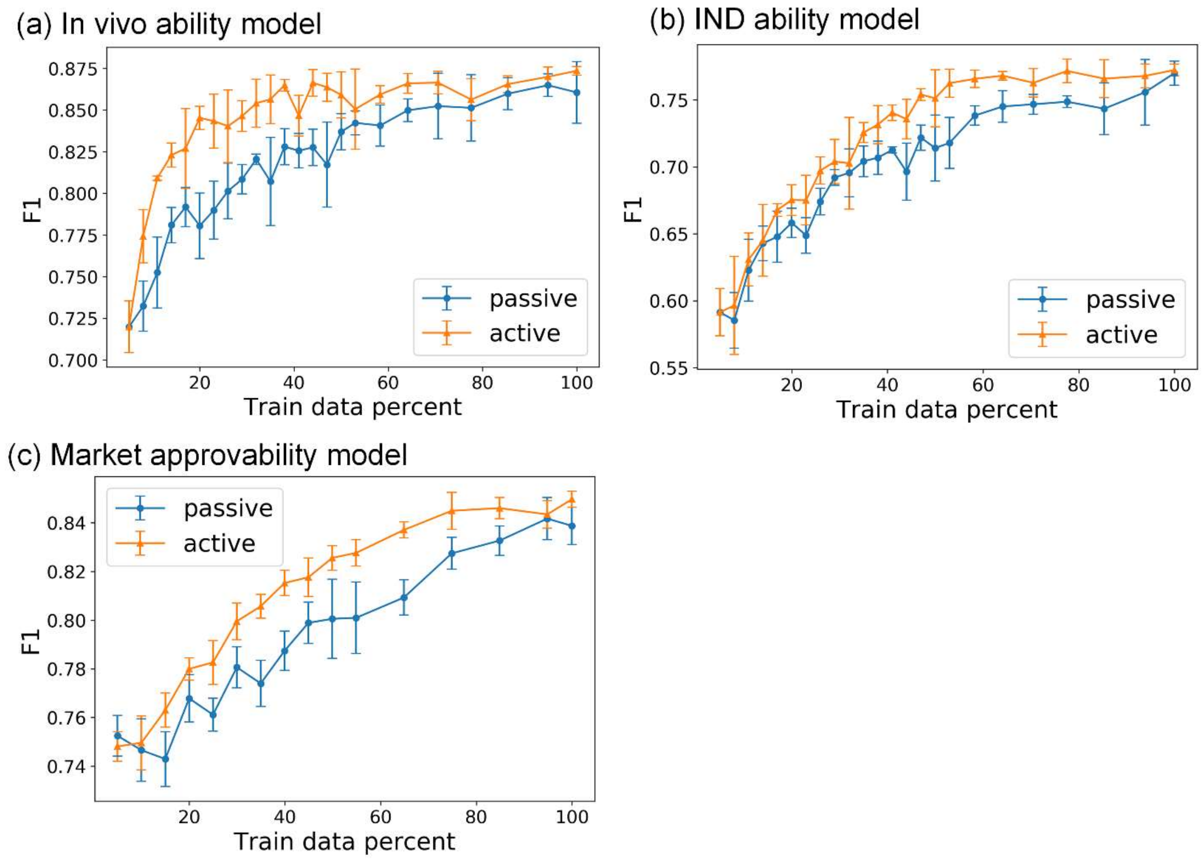

Figure S5. The F1 of models in the active learning process on test sets versus the percentage of training data used. (a) *In vivo* ability model (b) IND ability model (c) Market approvability model. The blue lines represent the performance of passive learning models, whereas the orange lines represent the performance of active learning models, and the error bar is the standard deviation of testing results in five-fold training.

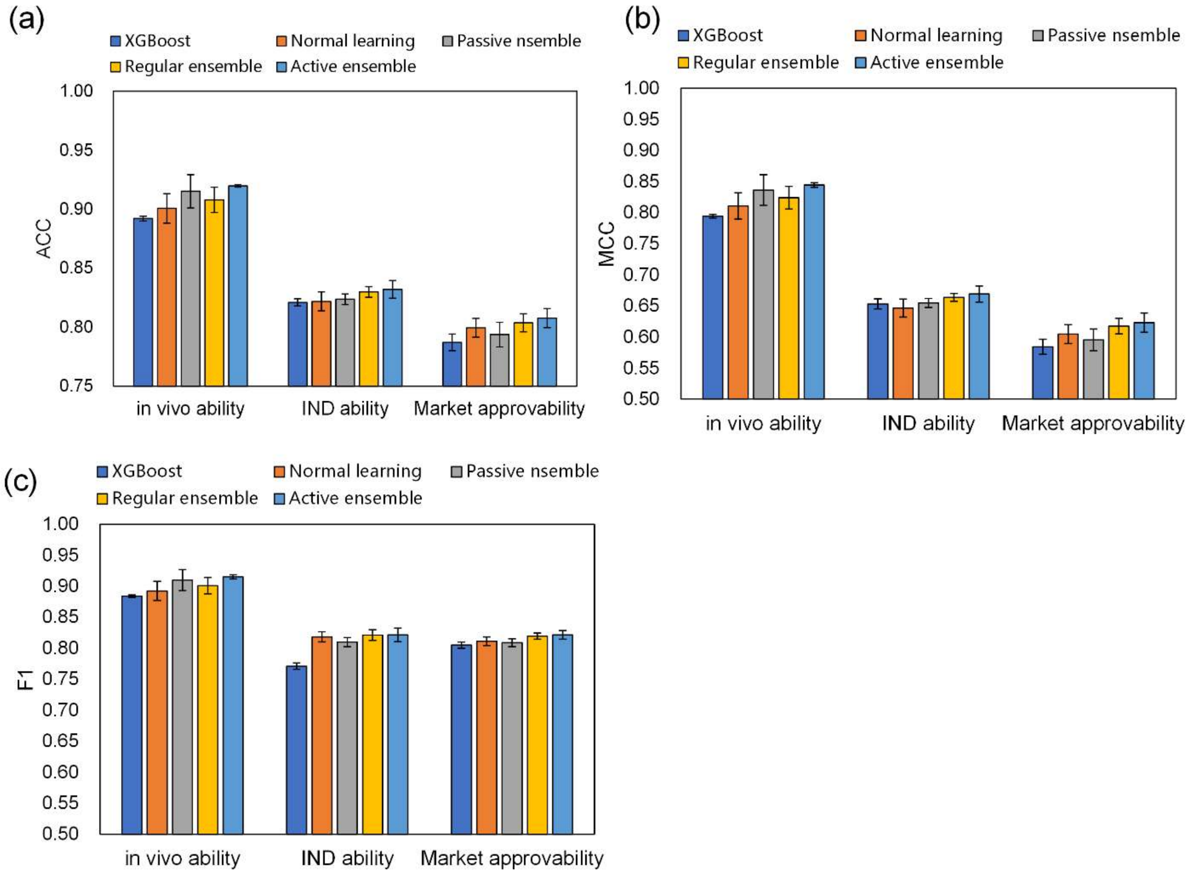

Figure S6. The performance of different methods on balanced test sets. (a) ACC (b) MCC (c) F1

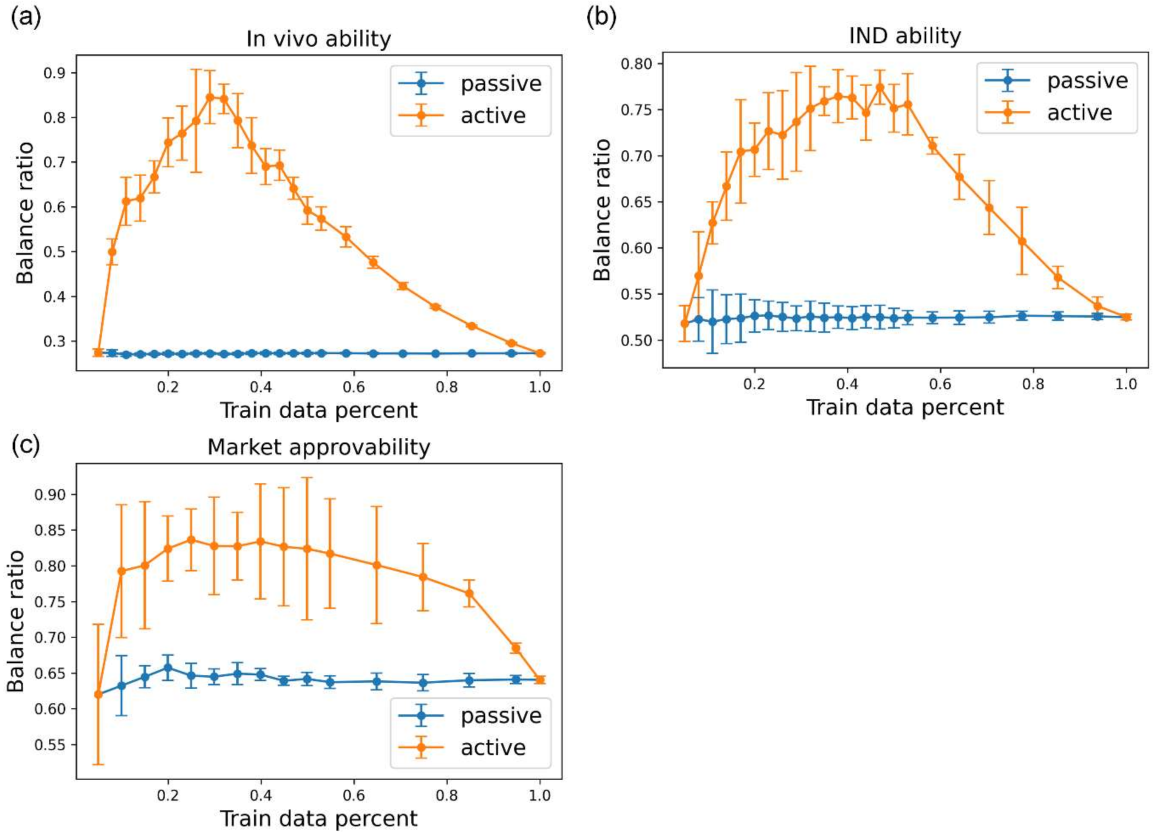

Figure S7. The balance ratio of training set in the active learning iterations. (a) *in vivo* ability model (b) IND ability model (c) Market approvability model

Table S1. Detailed information of DEKOIS2.0

Targets	Positive samples (positive samples after removing duplicates)	Negative samples (negative samples after removing duplicates)
11betaHSD1	40(39)	1200(1194)
17betaHSD1	40(23)	1199(1188)
A2A	40(36)	1200(1196)
ACE2	40(40)	1198(1197)
ACE	40(40)	1199(1197)
ACHE	40(40)	1196(1193)
ADAM17	40(33)	1200(1194)
ADRB2	40(40)	1200(1193)

AKT1	40(40)	1198(1197)
ALR2	40(35)	1199(1184)
AR	40(28)	1200(1194)
AURKA	40(33)	1200(1193)
AURKB	40(35)	1200(1192)
BCL2	40(40)	1199(1187)
BRAF	40(39)	1199(1192)
CATL	40(40)	1199(1195)
CDK2	40(36)	1200(1191)
COX1	40(37)	1197(1191)
COX2	40(40)	1199(1191)
CTSK	40(40)	1199(1188)
CYP2A6	40(37)	1198(1187)
DHFR	40(37)	1199(1191)
EGFR	40(35)	1200(1187)
EPHB4	40(36)	1200(1191)
ER-beta	40(36)	1200(1192)
ERBB2	40(37)	1196(1192)
FGFR1	40(33)	1198(1179)
FKBP1A	40(38)	1200(1196)
FXA	40(40)	1199(1190)
GBA	40(39)	1199(1195)
GR	40(36)	1199(1195)
GSK3B	40(37)	1200(1196)
HDAC2	40(34)	1200(1194)
HDAC8	40(30)	1199(1196)
HIV1PR	40(38)	1198(1188)
HIV1RT	40(38)	1200(1194)
HMGR	40(40)	1200(1191)
HSP90	40(31)	1200(1195)
IGF1R	40(38)	1198(1196)
INHA	40(27)	1200(1197)
ITK	40(39)	1200(1197)
JAK3	40(35)	1199(1196)
JNK1	40(35)	1200(1192)
JNK2	40(35)	1199(1192)
JNK3	40(30)	1200(1189)
KIF11	40(39)	1198(1196)
LCK	40(37)	1200(1194)
MDM2	40(40)	1196(1189)
MK2	40(38)	1198(1190)
MMP2	40(37)	1200(1193)
NA	40(40)	1191(1187)

P38-alpha	40(38)	1200(1193)
PARP-1	40(35)	1200(1193)
PDE4B	40(38)	1200(1197)
PDE5	40(40)	1200(1196)
PDK1	40(36)	1200(1198)
PI3Kg	40(36)	1200(1197)
PIM-1	40(36)	1199(1196)
PIM-2	40(39)	1200(1197)
PNP	40(40)	1198(1194)
PPARA	40(39)	1200(1199)
PPARG	40(39)	1197(1191)
PRKCQ	40(38)	1200(1198)
PR	40(38)	1199(1191)
PYGL-in	40(39)	1200(1199)
PYGL-out	40(40)	1199(1193)
QPCT	40(40)	1197(1195)
ROCK-1	40(38)	1198(1191)
RXR	40(40)	1197(1189)
SARS-HCoV	39(38)	1170(1159)
SIRT2	40(38)	1199(1190)
SRC	40(38)	1200(1196)
TIE2	40(35)	1199(1196)
TK	40(35)	1200(1195)
TPA	40(39)	1199(1198)
TP	40(32)	1197(1189)
TS	40(40)	1199(1194)
Thrombin	40(39)	1198(1197)
VEGFR1	40(35)	1200(1196)
VEGFR2	40(38)	1200(1195)
uPA	40(35)	1199(1193)

Table S2. The detailed information balance test data

Task	Positive or negative in datasets	Compound number
In vivo ability	Compounds in vivo testing stage (+)	2821
	Other compounds (-)	2821
IND ability	Compounds in clinical trials (+)	944
	Compounds in vivo testing stage (-)	944
Market approvability	Approved drugs (+)	381
	Compounds in clinical trials (-)	381

Table S3. The performance of different strategies on ChEMBL data

Target	Learning Method	ACC	MCC	F1
BRAF	Normal learning	0.956	0.535	0.977
	Passive ensemble	0.960	0.538	0.979
	Normal ensemble	0.960	0.557	0.979
	Active ensemble	**0.962**	**0.576**	**0.980**
DPP4	Normal learning	0.902	0.724	0.936
	Passive ensemble	0.912	0.744	0.944
	Normal ensemble	0.916	0.756	0.946
	Active ensemble	**0.917**	**0.762**	**0.946**
LCK	Normal learning	0.945	0.759	0.969
	Passive ensemble	0.948	0.774	0.971
	Normal ensemble	0.949	0.779	0.971
	Active ensemble	**0.950**	**0.784**	**0.972**
EGFR	Normal learning	0.933	0.512	0.964
	Passive ensemble	0.937	0.530	0.967
	Normal ensemble	0.938	0.536	0.967
	Active ensemble	**0.939**	**0.553**	**0.967**

The bold numbers represent the best results.

Table S4. The detailed information of investigational compounds and drugs from ChEMBL after removing duplicates and preprocessing

Investigational compounds	Drugs	All
1310	492	1802

Table S5. The performance of market approvability model on ChEMBL data

ACC	MCC	F1
0.728	0.383	0.569

Table S6. The virtual screening performance of *in vivo* ability model on DEKOIS 2.0

Target	AUC	EF1%	EF2%	EF5%	EF10%
11betaHSD1	0.743	2.6	3.8	2.6	3.1
17betaHSD1	0.912	39.1	28.3	13.9	7.8
A2A	0.796	5.6	5.6	7.2	5.3
ACE2	0.992	22.5	26.3	19.0	9.8
ACE	0.948	2.5	3.8	8.0	7.8
ACHE	0.763	0.0	1.3	2.0	3.0
ADAM17	0.957	27.3	24.2	14.5	8.2
ADRB2	0.900	10.0	6.3	7.0	5.5
AKT1	0.894	5.0	7.5	7.5	6.0
ALR2	0.883	2.9	4.3	6.9	6.0
AR	0.812	7.1	8.9	6.4	4.6
AURKA	0.875	18.2	13.6	7.3	5.8
AURKB	0.912	14.3	12.9	9.1	6.3
BCL2	0.681	0.0	0.0	1.0	1.3
BRAF	0.966	12.8	11.5	12.3	9.0
CATL	0.740	2.5	2.5	2.5	2.5
CDK2	0.849	11.1	8.3	6.1	5.6
COX1	0.861	8.1	5.4	4.9	4.6
COX2	0.962	20.0	15.0	13.0	8.8
CTSK	0.608	0.0	0.0	1.0	2.0
CYP2A6	0.692	5.4	8.1	3.8	2.7
DHFR	0.849	2.7	5.4	7.6	5.7
EGFR	0.900	14.3	11.4	9.7	6.6
EPHB4	0.914	8.3	11.1	10.6	7.5
ER-beta	0.928	11.1	12.5	10.0	7.2
ERBB2	0.935	5.4	10.8	10.8	7.0
FGFR1	0.923	3.0	9.1	7.3	6.7
FKBP1A	0.949	5.3	11.8	10.0	8.4
FXA	0.860	5.0	6.3	4.0	4.5
GBA	0.688	15.4	9.0	3.6	2.1
GR	0.956	8.3	6.9	7.8	9.2
GSK3B	0.841	10.8	9.5	7.6	4.9
HDAC2	0.939	14.7	13.2	11.8	8.2
HDAC8	0.980	26.7	20.0	15.3	10.0
HIV1PR	0.912	10.5	7.9	6.8	5.8
HIV1RT	0.809	5.3	3.9	4.7	3.7
HMGR	0.927	2.5	5.0	5.5	7.3

HSP90	0.904	0.0	4.8	6.5	5.8
IGF1R	0.955	10.5	17.1	11.1	7.9
INHA	0.527	7.4	5.6	2.2	1.5
ITK	0.830	10.3	6.4	3.6	3.6
JAK3	0.919	5.7	8.6	9.1	7.4
JNK1	0.827	11.4	11.4	9.1	5.7
JNK2	0.861	8.6	8.6	7.4	5.7
JNK3	0.875	6.7	11.7	9.3	6.7
KIF11	0.604	0.0	0.0	0.0	0.8
LCK	0.944	16.2	12.2	11.4	7.0
MDM2	0.888	0.0	3.8	5.5	5.3
MK2	0.930	15.8	13.2	7.4	5.8
MMP2	0.921	18.9	16.2	12.4	7.8
NA	0.827	0.0	0.0	0.0	2.3
P38-alpha	0.926	7.9	7.9	6.8	6.3
PARP-1	0.842	5.7	8.6	6.3	5.4
PDE4B	0.902	21.1	17.1	8.4	7.1
PDE5	0.915	5.0	6.3	9.0	7.0
PDK1	0.973	13.9	16.7	13.3	9.4
PI3Kg	0.836	5.6	5.6	5.0	4.4
PIM-1	0.853	8.3	9.7	6.1	4.4
PIM-2	0.890	12.8	9.0	7.2	6.2
PNP	0.915	10.0	7.5	6.5	5.5
PPARA	0.926	10.3	11.5	10.3	8.5
PPARG	0.914	15.4	12.8	9.2	6.7
PRKCQ	0.936	18.4	17.1	11.1	7.6
PR	0.919	15.8	14.5	10.0	6.3
PYGL-in	0.848	5.1	5.1	6.7	3.8
PYGL-out	0.873	0.0	3.8	3.5	3.0
QPCT	0.659	0.0	1.3	1.5	1.5
ROCK-1	0.812	10.5	10.5	5.8	4.7
RXR	0.980	27.5	22.5	14.5	9.0
SARS-HCoV	0.465	0.0	0.0	0.5	1.1
SIRT2	0.756	5.3	5.3	3.2	2.1
SRC	0.872	15.8	11.8	8.9	6.6
TIE2	0.927	14.3	14.3	12.0	7.7
TK	0.809	2.9	4.3	5.7	3.7
TPA	0.898	5.1	5.1	7.7	5.9
TP	0.852	0.0	0.0	1.9	2.8
TS	0.890	0.0	6.3	5.0	5.8
Thrombin	0.819	0.0	3.8	5.6	4.1
VEGFR1	0.872	17.1	12.9	8.6	5.4
VEGFR2	0.895	10.5	11.8	8.9	6.8

uPA	0.853	5.7	7.1	6.9	5.4
**mean**	**0.860**	**9.2**	**9.1**	**7.4**	**5.6**
**median**	**0.890**	**8.1**	**8.6**	**7.3**	**5.8**

Table S7 The detailed information of bioactivity data

	Training		Test	
Targets	Active	Inactive	Active	Inactive
BRAF	1880	73	206	10
DPP4	1182	378	135	38
LCK	1519	183	162	27
EGFR	5924	529	655	62
